# A self‐help intervention for reducing time to diagnosis in Indonesian women with breast cancer symptoms

**DOI:** 10.1002/pon.5316

**Published:** 2020-01-06

**Authors:** Hari Setyowibowo, Joke A. M. Hunfeld, Aulia Iskandarsyah, Whisnu Yudiana, Jan Passchier, Sawitri S. Sadarjoen, Dharmayanti F. Badudu, Drajat R. Suardi, Edith van‘t Hof, Marit Sijbrandij

**Affiliations:** ^1^ Department of Clinical, Neuro‐ and Developmental Psychology Vrije Universiteit Amsterdam Amsterdam The Netherlands; ^2^ Department of Educational Psychology, Faculty of Psychology Universitas Padjadjaran Jatinangor Indonesia; ^3^ Department of Psychiatry, Section Medical Psychology and Psychotherapy Erasmus MC University Medical Center Rotterdam The Netherlands; ^4^ Department of Clinical Psychology, Faculty of Psychology Universitas Padjadjaran Jatinangor Indonesia; ^5^ Department of Experimental Psychology, Faculty of Psychology Universitas Padjadjaran Jatinangor Indonesia; ^6^ Department of Surgical Oncology Hasan Sadikin Hospital Bandung Indonesia

**Keywords:** adherence, breast, cancer, cluster randomized controlled trial, diagnosis, health education, Indonesia, oncology, self‐help psychoeducation

## Abstract

**Objective:**

We investigated the effectiveness of a self‐help intervention named PERANTARA, which aims to improve adherence to diagnostic procedures among women with breast cancer (BC) symptoms to reduce the time to a definitive diagnosis.

**Methods:**

With a cluster randomized crossover design across four hospitals, PERANTARA and treatment as usual (TAU) or TAU only was provided at successive periods in a randomly determined order. The main outcome was the time between the first medical consultation and the definitive diagnosis. Secondary outcomes were BC knowledge, measured by the Breast Cancer Knowledge Test (BCKT); symptoms of anxiety and depression, measured by the Hospital Anxiety and Depression Scale (HADS); quality of life, measured by the World Health Organization Quality of Life‐BREF (WHOQOL‐BREF); and health status, measured by the EQ‐5D‐5L. A linear mixed model analysis was conducted to analyse the outcomes.

**Results:**

We recruited 132 women with BC symptoms from four hospitals; 67 participants were in the intervention group, and 65 participants were in the control group. PERANTARA reduced the time to definitive diagnosis by 13.3 days (*M* [*SD*]: 25.90 [23.20] in the intervention group vs 39.29 [35.10] in the control group; mean difference = −13.26, 95% CI = −24.51 to −2.00, *P* = .02). No significant difference was found between the groups in BC knowledge, symptoms of anxiety, depression, quality of life, or health status.

**Conclusions:**

PERANTARA reduced the time to definitive diagnosis among Indonesian women with BC symptoms. Psychoeducation may be an important addition to regular BC care to prevent undue delays in diagnostic procedures.

## 1 BACKGROUND

Breast cancer (BC) is a commonly diagnosed cancer and the leading cause of cancer mortality among women in low‐ and middle‐income countries (LMICs), including Indonesia, where resources for prevention, diagnosis, and treatment are limited.^1^ In the clinical BC management, an accurate and timely diagnosis is critical.^2^

In Indonesia, over 80% of BC cases are found at an advanced stage.^3^ When visiting the hospital to check for breast abnormalities, approximately 67% of women do not pursue a definitive diagnosis.^4^ Women with BC who delayed hospital visits for treatment were found to distrust medical procedures because of a perceived lack of information regarding their positive effects.^5^ In contrast, many patients prefer visiting alternative healers who are considered to be more supportive, inexpensive, and effective.^5^, ^6^ To improve access and facilitate early diagnosis, interventions for women with early BC symptoms should focus on addressing these barriers to reduce diagnosis delay.

A cross‐sectional study among 70 women with BC in Indonesia showed that 41% to 86% were not satisfied with information about BC that they received.^7^ The provision of information about BC symptoms, diagnosis, and treatment through health education^8^ and support in coping with psychosocial issues (psychoeducation) was useful for promoting health behaviour changes and improving BC‐related knowledge, reducing anxiety and depression, and improving quality of life in women with BC symptoms or BC survivors.^9^, ^10^, ^11^, ^12^ However, no studies have been conducted on the use of self‐help interventions consisting of health and psychoeducation to encourage women with BC symptoms to receive timely diagnoses.

We developed and evaluated a culturally sensitive, narrative self‐help intervention named PERANTARA (PEngantar peRAwataN kesehaTAn payudaRA, translated as introduction to breast health treatment,^13^ that aims to motivate women with BC symptoms to comply with diagnostic procedures. PERANTARA consists of health education and psychoeducation and uses a narrative strategy, which involves the use of testimonials and storytelling.^14^ This strategy is acceptable for patients with low health literacy for communication of BC‐related information.^15^, ^16^, ^17^, ^18^, ^19^

The primary aim of this study was to investigate the effectiveness of PERANTARA in reducing the time (in days) from the first consultation with a doctor for BC symptoms to the time of a definitive diagnosis. The secondary aims were to examine the effects of PERANTARA on BC knowledge, symptoms of anxiety and depression, quality of life, and health status.

## 2 METHODS

The study protocol has been published elsewhere.^20^ The study was approved by the Health Research Ethics Committee of Dr. Hasan Sadikin General Hospital in Bandung on 23 December 2013 (Document No: LB.04.01/A05/EC/127/XII/2013).

### 2.1 Study design and participants

We used a cluster randomized crossover design in which four hospitals in Bandung, West Java, Indonesia, provided either PERANTARA plus treatment as usual (TAU) or TAU only to participants in a randomly determined order (Appendix 1). Two predefined periods were determined (Appendix 2). In the first period (January 2017‐May 2017), two hospitals were allocated to the intervention group (PERANTARA plus TAU), and two hospitals were allocated to the control group (TAU only). In the second period (February 2017‐September 2017), the two hospitals allocated to the intervention group were assigned to control, and vice versa. Randomization was performed by a team member who was not involved in the data collection.

The inclusion criteria were as follows: (a) newly admitted female outpatients who visited the hospitals with BC symptoms before obtaining a definitive diagnosis; (b) age 18 years and older; (c) adequate command of the Indonesian language; and (d) no previous psychiatric consultations, as determined by medical records. Power calculations suggested a minimum sample size of 41 participants per group (power = 0.80, alpha = 0.05 two‐sided).^20^ To account for 30% attrition at follow‐up, we aimed to include at least 106 participants (53 per group).

### 2.2 Procedures

Eligible patients were asked to provide oral and written informed consent by research assistants with bachelor's degrees in psychology. After the baseline assessment (T0), the participants in the intervention group received PERANTARA and TAU, whereas those in the control group received TAU. For the intervention group, the research assistants provided a brief explanation and instruction about PERANTARA. This group was then requested to view and read the PERANTARA materials within 7 days following the baseline assessment. The post‐intervention assessment (T1) took place 7 days after the intervention, and the follow‐up assessment (T2) was scheduled for 3 months (12 weeks) following T1.

### 2.3 Intervention

PERANTARA is a self‐help intervention that combines printed and audio‐visual health education and psychoeducation materials.^13^, ^20^ The printed material covers three core themes: (a) “What is in my breast?,” providing a brief explanation of BC symptoms to promote an accurate understanding of BC and consulting a doctor as a credible source; (b) “Why should you immediately consult a doctor?,” offering a brief explanation of breast examination procedures to raise awareness about BC symptoms and increases motivation to follow diagnostic procedures; and (c) “You are not alone,” recommending to seek support from significant persons and institutions. The audio‐visual material consists of a DVD that provides the testimonials and stories of two BC survivors who encourage patients to engage in active coping and seek social support and to follow medical procedures. A pilot study showed that the prototype was feasible and acceptable.^13^ See Appendix 3.

### 2.4 Treatment as usual

TAU for women with BC symptoms in the four study hospitals consists of consultations with an oncologist about medical examination procedures and an educational poster on the wall in the hospital waiting room. Psychosocial services are usually not provided.

### 2.5 Measures

The background characteristics included age, marital status, education level, income level, travel time to the hospital, insurance status, and consultation with a traditional healer.

Time to diagnosis, defined as the time between the first consultation with a doctor for BC symptoms to the time (in days) of a definitive diagnosis, was assessed using the following interview questions: (a) What was the date that you consulted a doctor in the hospital regarding your BC symptoms? (b) What was the date that you received a definitive diagnosis? To identify whether the cause of the delay was due to the patient or the doctor, we asked the following questions: (a) On which date did your doctor schedule the examination (to make the definitive diagnosis)? (b) On which date did your doctor schedule a consultation to provide the definitive diagnosis? To verify the interview results, we compared the participants' responses with both the individuals' and hospitals' medical records.

BC knowledge was assessed using the Indonesian version of the Breast Cancer Knowledge Test (BCKT), a 20‐item questionnaire that consists of two subscales: (a) general knowledge (12 items) and (b) curability (eight items).^5^, ^21^ In this study, only the curability subscale had acceptable reliability (Cronbach's coefficients of *α* = .54 at T0, *α* = .52 at T1, and *α* = .69 at T2). Therefore, we decided to use only the curability subscale (score range of 0‐8, with higher scores indicative of more knowledge about BC curability).

The 14‐item, self‐report Hospital Anxiety and Depression Scale (HADS) measured symptoms of anxiety and depression during the past week.^22^, ^23^ The HADS consists of two subscales: anxiety (HADS‐A, 7 items, score range of 0‐21) and depression (HADS‐D, 7 items, score range of 0‐21). Higher scores indicate a higher symptom level.

The Indonesian version of the 26‐item World Health Organization Quality of Life‐BREF (WHOQOL‐BREF)^24^, ^25^, ^26^ was used to measure quality of life during the past 4 weeks. Two items measure quality of life (score range of 0‐100) and health satisfaction in general (score range of 0‐100). Twenty‐four items measure four broad domains, namely, physical health (seven items), psychological health (six items), social relationships (three items), and environment (eight items). Higher scores indicate better quality of life.

The Bahasa Indonesia version of the EQ‐5D‐5 L measured health status. The EQ‐5D‐5L defines health in relation to five dimensions: mobility (MO), self‐care (SC), usual activities (UA), pain/discomfort (PD), and anxiety/depression (AD), with five levels per dimension: (a) no problems, (b) slight problems, (c) moderate problems, (d) severe problems, and (e) extreme problems/unable. A single value indicates the level selected for each dimension. The second part is a visual analogue scale of overall health status (EQ‐VAS), with scores ranging from 0 (“the worst health you can imagine”) to 100 (“the best health you can imagine”). The EQ‐5D‐5L has been shown to be valid and reliable when used in Indonesia.^27^, ^28^

### 2.6 Data analysis

We used chi‐square tests and independent samples *t* tests in SPSS version 24 to compare baseline demographic characteristics between the intervention and control groups and between participants who discontinued and who completed the study.

Outcome data were analysed using linear mixed models in R version 1.1.423 with the Lme4 package.^29^, ^30^ We followed the recommended procedures for multilevel modelling.^31^ An advantage of mixed model analyses is that the full data set is used, including missing data.^32^ A two‐level model was used to analyse the primary outcome (level 1: participant and level 2: hospital), and a three‐level model (level 1: measurement time points [MTPs]; level 2: participant; and level 3: hospital) was used to analyse the secondary outcomes. A generalized mixed model was employed with treatment, MTPs, and the interaction between PERANTARA and MTPs as fixed effects and hospital and subject as random effects. The difference in means between the two groups (intervention and control group) at each MTP and the 95% CI was derived from the generalized mixed model. The effect size was calculated by subtracting the group means and dividing the result by the standard deviation of the population from which the groups were sampled. All analyses were described and agreed upon in the statistical analysis plan before unmasking the study (Appendix 4).

## 3 RESULTS

### 3.1 Flow and characteristics of the participants

We approached 185 eligible participants, of whom 132 (71.4%) consented to participate (67 in the intervention group and 65 in the control group). For the primary outcome, we analysed the data of 107 participants, of whom 51 were in the intervention group and 56 were in the control group. For the secondary outcomes, we assessed 67 participants in the intervention group and 65 participants in the control group at the baseline assessment (T0). At T1, the follow‐up rates were 80.6% (54/67) in the intervention group and 76.9% (50/65) in the control group, and at T2, they were 52.2% (35/67) in the intervention group and 61.5% (40/65) in the control group. The difference in attrition between the primary and secondary outcomes was due to the primary outcome being based on both the interview and the hospital medical records (n = 107), whereas the secondary outcomes were based on the self‐report instruments returned by 132 participants. No serious adverse events were reported in either group.

There were no significant differences found in background characteristics between the intervention and control groups (Table 1). In addition, background characteristics did not differ between the participants who discontinued participation and those who completed the study.

**Table 1:** Baseline characteristics of the intervention and control groups and the results of the tests of differences (total n = 132)

Characteristics	Intervention Group (n = 67)	Control Group (n = 65)	*X* or *t*	*df*	*P* value*
Age, mean (*SD*)	38.04 (11.89)	37.92 (14.65)	−0.05	130	.96
Education, n (%)			2.66	2	.26
Basic	8 (11.90)	14 (21.60)			
Middle	44 (65.70)	35 (53.80)			
High	15 (22.40)	16 (24.60)			
Hospital, n (%)			0.14	3	.99
Hospital A	20 (29.90)	21 (32.40)			
Hospital B	9 (13.30)	9 (13.80)			
Hospital C	20 (29.90)	19 (29.20)			
Hospital D	18 (26.90)	16 (24.60)			
Income, n (%)					
<2 million Rupiah	28 (41.80)	32 (49.30)	2.24	2	.33
2‐4 million Rupiah	27 (40.30)	27 (41.50)			
>4 million Rupiah	12 (17.90)	6 (9.20)			
Location/Residence			0.30	1	.58
Urban	31 (46.3)	27 (41.5)			
Rural	36 (53.7)	38 (58.5)			
Time to hospital, n (%)			0.09	1	.83
Less than an hour	55 (81.10)	52 (82.10)			
Between 2 and 3 h	12 (18.90)	13 (17.90)			
Alternative medicine n (%)			0.86	1	.41
No	54 (80.60)	48 (73.80)			
Yes	13 (19.40)	17 (26.20)			
Breast cancer knowledge test (BCKT)					
Curability, mean (*SD*)	5.45 (1.74)	5.34 (1.76)	−0.36	130	.72
Anxiety and Depression Symptoms (HADS)					
Anxiety (HADS‐A), mean (*SD*)	7.75 (3.43)	7.66 (4.37)	−0.12	130	.90
Depression (HADS‐D)	4.94 (3.02)	5.25 (3.35)	0.55	130	.58
Quality of Life (WHOQOL‐BREF)					
Physical health, mean (*SD*)	64.60 (14.49)	61.70 (13.28)	−1.20	130	.23
Psychological, mean (*SD*)	63.30 (15.33)	61.73 (16.06)	−0.58	130	.57
Social relationships, mean (*SD*)	63.05 (13.77)	62.56 (15.10)	−0.20	130	.84
Environment, mean (*SD*)	60.16 (13.03)	58.36 (12.14)	−0.82	130	.41
Health Status (EQ‐5D‐5L)					
Index score, mean (*SD*)	0.77 (0.20)	0.74 (0.27)	−0.81	130	.42
Visual analogue score, mean (*SD*)	71.30 (16.80)	66.91 (22.76)	−1.27	130	.21

*Note*: Chi‐square test for nominal variables and independent samples *t* tests for continuous variables.

* Significant at *P* < .05.

### 3.2 Outcomes

#### 3.2.1 Primary outcome (time to diagnosis)

The linear mixed model analysis (Table 2) showed a significantly larger reduction in time (days) to diagnosis in the intervention group (*M* = 25.90, *SD* = 23.20) than in the control group (*M* = 39.29, *SD* = 35.10). PERANTARA reduced the time between the first visit to the hospital and provision of a definitive diagnosis by an average of 13.3 (*SE* = 5.67; 95% CI = −24.51 to −2.00, *P* = .02) days. The effect size of the between‐group difference approached medium (Cohen's *d* = .43).

**Table 2:** Statistics and test results for the primary and secondary outcomes

Outcomes	Measurement Time	Descriptive Statistics				
		*M* (*SD*)		Mixed Model Analysis		
		Intervention Group	Control Group	Difference in LS mean (95% CI)	*P* value*	Effect size^a^
Time to diagnosis		25.90 (23.20)	39.29 (35.10)	−13.26 (−24.51 to −2.00)	.02	0.43
Breast Cancer Knowledge Test (BCKT)						
Curability	T1	5.81 (1.67)	5.64 (1.61)	0.04 (−0.58 to 0.67)	.88	ns
scale (0‐8)	T2	5.69 (1.99)	5.08 (1.97)	0.61 (−0.09 to 1.33)	.09	ns
Anxiety and Depression Symptoms (HADS)						
Anxiety (HADS‐A)	T1	7.04 (3.77)	6.42 (4.16)	0.83 (−0.42 to 2.10)	.19	ns
scale (0‐21)	T2	5.23 (3.91)	5.73 (3.70)	−0.43 (−1.89 to 1.01)	.55	ns
Depression (HADS‐D)	T1	4.69 (3.35)	5.16 (3.69)	−0.17 (−1.29 to 0.94)	.76	ns
scale (0‐21)	T2	3.86 (3.21)	4.28 (3.11)	0.13 (−1.14 to 1.42)	.83	ns
Quality of life (WHOQOL‐BREF)						
Physical health	T1	62.63 (13.37)	62.50 (12.46)	−1.87 (−6.37 to 2.62)	.41	ns
scale (0‐100)	T2	68.67 (11.19)	67.05 (12.80)	−0.27 (−5.47 to 4.92)	.92	ns
Psychological	T1	62.34 (11.97)	63.00 (12.92)	−1.56 (−6.02 to 2.90)	.49	ns
scale (0‐100)	T2	66.19 (14.17)	66.25 (13.16)	−2.08 (−7.13 to 2.95)	.41	ns
Social relationships	T1	61.72 (12.69)	63.33 (13.25)	−2.13 (−6.15 to 1.89)	.3	ns
scale (0‐100)	T2	64.28 (14.93)	64.37 (14.24)	−1.31 (−5.95 to 3.31)	.57	ns
Environment	T1	59.31 (13.37)	56.68 (10.77)	1.29 (−1.75 to 4.34)	.4	ns
scale (0–100)	T2	63.92 (12.73)	61.48 (12.92)	0.13 (−3.33 to 3.61)	.94	ns
Health status (EQ‐5D‐5 L)						
Index score	T1	0.82 (0.15)	0.80 (0.20)	0.00 (−0.05 to 0.07)	.77	ns
scale (0‐1)	T2	0.90 (0.21)	0.84 (0.17)	0.04 (−0.02 to 0.11)	.22	ns
Visual analogue score	T1	77.87 (13.76)	77.88 (16.58)	−0.25 (−5.59 to 5.09)	.93	ns
scale (0‐100)	T2	85.63 (11.10)	80.45 (14.42)	4.57 (−1.57 to 10.7)	.14	ns

*Note*: The mixed model included treatment, time, and the interaction between treatment and visit as fixed effects, the baseline outcome measurement as the covariate, and the hospital and subject as random effects.

* Significant at *P* < .05.

a The effect size was calculated by dividing the LS mean by the SD.

#### 3.2.2 Secondary outcomes

##### BC knowledge

The linear mixed models showed no significant difference in BCKT curability between the intervention and control groups at T1 (*M* [*SD*] 5.81 [1.67] vs 5.64 [1.61], *P* = .88) or at T2 (*M* [*SD*] 5.69 [1.99] vs 5.08 [1.97], *P* = .09).

##### Symptoms of anxiety and depression

At T1, we found no significant difference between the intervention and control groups in HADS anxiety (*M* [*SD*] 7.04 [3.77] vs 6.42 [4.16], *P* = .19) or HADS depression (4.69 [3.35] vs 5.16 [3.69], *P* = .76) score. At T2, there was also no significant difference between the intervention and control groups in HADS anxiety (*M* [*SD*] 5.23 [3.91] vs 5.73 [3.70], *P* = .55) or HADS depression (3.86 [3.21] vs 4.28 [3.11], *P* = .83) score.

##### Quality of life

The linear mixed model analysis on the WHOQOL‐BREF scores showed no significant difference between the intervention and control groups at T1 in the physical health (*M* [*SD*] 62.63 [13.37] vs 62.50 [12.46], *P* = .41), psychological (62.34 [11.97] vs 63.00 [12.92], *P* = .49), social relationships (61.72 [12.69] vs 63.33 [13.25], *P* = .3), or environment (59.31 [13.37] vs 56.68 [10.77], *P* = .4) domain score. At T2, there was also no significant difference between the intervention and control groups in the physical health (68.67 [11.19] vs 67.05 [12.80], *P* = .92), psychological (66.19 [14.17] vs 66.25 [13.16], *P* = .41), social relationships (64.28 [14.93] vs 64.37 [14.24], *P* = .57), or environment (63.92 [12.73] vs 61.48 [12.92], *P* = .94) domain score.

##### Health status

The linear mixed model analysis showed no significant difference between the intervention and control groups at T1 in the EQ‐5D‐5L index score (*M* [*SD*] 0.82 [0.15] vs 0.80 [0.20], *P* = .77) or the visual analogue score (77.87 [13.76] vs 77.88 [16.58], *P* = .93). At T2, no significant difference between the groups was found in the EQ‐5D‐5L index score (0.90 [0.21] vs 0.84 [0.17], *P* = .22) or the visual analogue score (85.63 [11.10] vs 80.45 [14.42], *P* = .14).

## 4 DISCUSSION

The primary aim of the current study was to evaluate the effect of PERANTARA on the time between the first consultation at the hospital regarding BC symptoms and the provision of a definitive diagnosis among Indonesian women with early BC symptoms. The results of this study confirm that PERANTARA had a small to medium effect (Cohen's *d* = .43) in reducing the time to diagnosis by an average of 13.3 days, for an average of 27 days between the first consultation and the definitive diagnosis. PERANTARA had no significant effect on the secondary outcomes, that is, knowledge of BC curability, symptoms of anxiety and depression, quality of life, and health status.

Our study was the first evaluating a self‐help intervention to reduce the time between the first consult with a doctor and a definitive diagnosis for women with BC symptoms in an under‐resourced LMIC setting such as Indonesia. Our findings are in line with previous studies showing that self‐help interventions promote health behaviour.^10^, ^12^ However, we did not find reductions in anxiety and depression symptoms or improvements in quality of life or health status comparing the intervention and control groups. This seems remarkable since previous studies in Turkey and Taiwan found beneficial effects of psychoeducation on anxiety, depression, and quality of life.^11^, ^12^ A plausible explanation is that PERANTARA lacks specific guidance on how to adequately deal with distress to improve daily functioning and quality of life.

### 4.1 Study limitations

The limitations of the current study were that the four hospitals were located in an urban setting and thus may have different characteristics than rural hospitals in Indonesia. Another limitation was the ethnic homogeneity of the study sample, which may limit the generalizability of our findings to other populations. Further, we used only the time interval in days as our primary outcome. This measure was chosen because of its feasibility and since the passage of time is the crucial determining factor for tumour growth. However, we have only limited information concerning the reasons for the delay and whether the delay was caused by the hospital or the patient herself.

### 4.2 Clinical implications

In conclusion, our findings provide evidence that PERANTARA can be used to encourage women with BC symptoms to promptly consult an oncologist and follow diagnostic procedures. This is important since research has shown that Indonesian women with early BC symptoms usually wait to visit a hospital until the disease is already in an advanced stage^33^ or do not adhere to diagnostic procedures,^4^ which negatively affects BC prognosis. The standards of the National Health Service of England for waiting times for suspected and diagnosed cancer patients^34^ suggest a maximum of 2 weeks before seeing a specialist for further diagnostic follow‐up for all patients with suspected BC symptoms referred by general practitioners. In our study, PERANTARA was able to reduce the time to an average of 27 days. Since this period is still almost 2 weeks longer than recommended, there is still room for improvement. Nevertheless, our results underline that with relatively low effort, it is possible to significantly improve adherence to diagnostic procedures for BC. Further cultural adaptation of the PERANTARA programme for other areas of Indonesia and for other LMIC settings is recommended.

### 4.3 Research implications

Future research could focus on the effectiveness of the different elements of the PERANTARA and on different delivery formats, such internet, mobile phones, face‐to‐face (group or individual), or a blended version of both self‐help and health care intervention. Furthermore, it is important to adapt PERANTARA to other BC populations inside and outside of Indonesia, taking into account the language, culture, and context to guarantee that it is compatible with local cultural patterns, meanings and values.^35^

## CONFLICT OF INTEREST

The authors have no conflicts to declare.

## CLINICAL TRIAL REGISTRATION NUMBER

ISRCTN12570738.

## Supporting information


**Appendix** S1: CONSORT checklist.

**Appendix** S2: CONSORT diagram to illustrate data completeness related to primary and secondary outcomes

**Appendix** S3: Description of the intervention

**Appendix** S4: Statistical analysis plan

## Data Availability

The data that support the findings of this study are available on request from the corresponding author.
