# Supplementary material for: A self‐help intervention for reducing time to diagnosis in Indonesian women with breast cancer symptoms
Source: Psychooncology. 2020 Jan 6;29(4):696–702. doi: 10.1002/pon.5316 (PMC7217183; doi:10.1002/pon.5316)

Appendix 2

CONSORT diagram to illustrate data completeness related to primary and secondary outcomes.

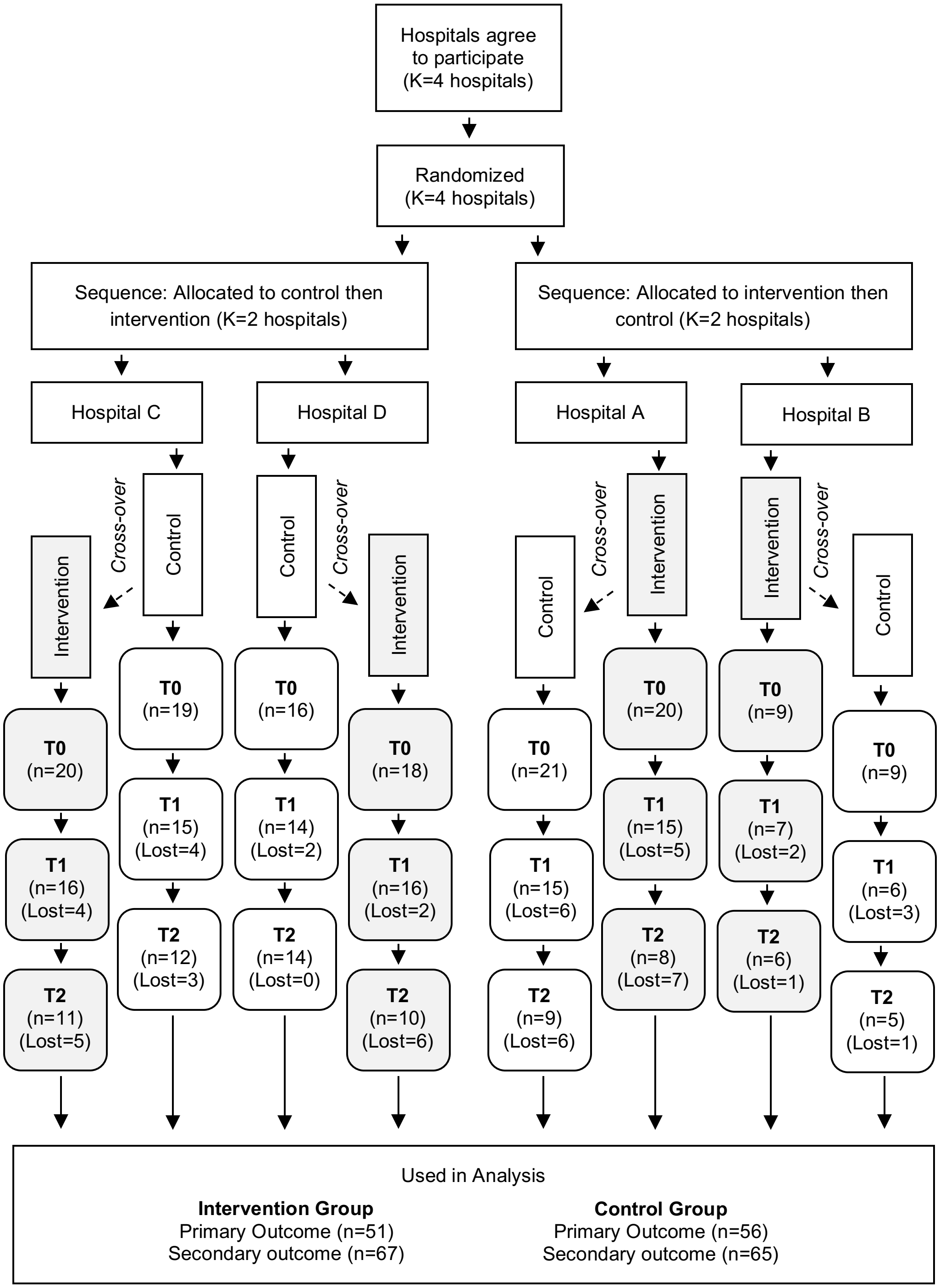

Supplement: Supplementary file 2 — Appendix S2: CONSORT diagram to illustrate data completeness related to primary and secondary outcomes [file PON-29-696-s002.pdf]
