# Supplementary material for: A self‐help intervention for reducing time to diagnosis in Indonesian women with breast cancer symptoms
Source: Psychooncology. 2020 Jan 6;29(4):696–702. doi: 10.1002/pon.5316 (PMC7217183; doi:10.1002/pon.5316)
Supplement: Supplementary file 3 — Appendix S3: Description of the intervention [file PON-29-696-s003.docx]

**Appendix 3. Description of the Intervention**

PERANTARA is a culturally sensitive, narrative self-help intervention. The Indonesian word ‘perantara’ means ‘mediator’; the PERANTARA acronym is derived from PEngantar peRAwataN kesehaTAn payudaRA (“introduction to breast health treatment”). PERANTARA is a self-help intervention that combines printed and audio-visual material.

1. **PRINTED MATERIAL**

The printed material includes a flipchart that provides a brief explanation of breast cancer knowledge (symptoms, diagnosis and treatment procedures, etc.) and the importance of social support to motivate women with breast cancer symptoms to receive a timely diagnosis.

**Title**

PERANTARA: SEGERA PERIKSA KE DOKTER

(PERANTARA: CONSULT YOUR DOCTOR IMMEDIATELY)

**Main Themes**

1. What is in my breast? A brief explanation of breast cancer symptoms to enable patients to have an accurate understanding of breast cancer and motivation to seek information from a doctor as a credible source
2. Why should you immediately consult a doctor? A brief explanation of breast examination procedures to raise awareness of BC symptoms and increase motivation to follow diagnostic procedures
3. You are not alone. Emphasizes the importance of social support and informs patients that many people around them might provide practical or emotional support

Additional:

Personal notes. A space for women with breast cancer symptoms to record important things, for example, complaints, questions asked, doctor's advice and other important notes

**B. AUDIO-VISUAL MATERIAL**

A DVD that provides the testimonials and stories of two BC survivors who share their experiences, encourages patients to engage in active coping and seek social support and recommend that individuals consult a doctor immediately when they discover abnormalities in the breast and that they follow the medical procedures.

**Title**

KISAH NYATA: DUA PEREMPUAN TANGGUH

(THE STORY OF TWO WARRIORS)

**Key Messages**

1. Introduction.

Sample script:

“This is a true story of the struggles of two tough women since they discovered abnormalities in their breasts until they successfully underwent various challenges during the treatment process. Now, they are still actively working for their community and beloved families.”

1. Reduce delay.

Sample script:

“If we find things that are suspicious or anything different than usual, we consult with a doctor or other health professionals to confirm what the problem is...”

“Do not be too scared at first because not all lumps are cancer. The important thing is to consult the doctor immediately to know the examination procedures.”

1. Coping with psychosocial issues.

Sample script:

“...When you go through the process of examination and treatment, feelings of sadness and loneliness will probably arise. You do not have to worry too much because you are not alone. There are many people who care about you! Ask your husband, family or friends to accompany you. Share stories, feelings and expectations about things that need to be done...”

“...My message to families, especially to women’s husbands, is that emotional support for a spouse or someone who is undergoing examination or treatment for breast cancer is very important. Without the support of their husbands, their spirit will fall."

1. Contacts.

Important list of sources of informational, financial and emotional support.
