# Supplementary material for: A self‐help intervention for reducing time to diagnosis in Indonesian women with breast cancer symptoms
Source: Psychooncology. 2020 Jan 6;29(4):696–702. doi: 10.1002/pon.5316 (PMC7217183; doi:10.1002/pon.5316)
Supplement: Supplementary file 4 — Appendix S4: Statistical analysis plan [file PON-29-696-s004.docx]

**Appendix 4**

**STATISTICAL ANALYSIS PLAN**

1. Compare the demographic characteristics of the intervention group and control group at baseline. Use the chi-square test for nominal variables and independent samples t-tests in SPSS version 24 for continuous variables.
2. For the primary outcome, apply a two-level model to analyse the data (Level 1: participant and Level 2: hospital). Employ a generalized mixed model with PERANTARA as a fixed effect and hospital as a random effect.
3. For the secondary outcomes, apply three-level models (Level 1: measurement time points (MTPs); Level 2: participant; and Level 3: hospital). Employ a generalized mixed model with the interaction between PERANTARA and MTPs as fixed effects, a baseline measurement as the covariate, and hospital and subject as random effects.
4. Note: The difference in means between the two groups (intervention and control group) at each measurement time point (T0, T1, and T2) and the 95% CI were derived from the generalized mixed model. The effect size was calculated by subtracting the mean of one group from that of the other and dividing the result by the standard deviation of the population from which the groups were sampled.

**R SCRIPTS**

Primary Outcome

Outcome: Time to Diagnosis (TD)

Level 1: Participants

Level 2: Hospital

# Null Model

Model1.1H <- lme(fixed = TD~1, random = ~1|Hospital , data = data, method = "ML",na.action=na.omit)

summary(Model1.1H)

#add fixed effect

Model2.1H <- lme(fixed = TD~ PERANTARA,

random = ~1|Hospital , data = Data, method = "ML", na.action=na.omit)

summary(Model2.1H)

#deciding Best Model

anova(Model1.1H,Model2.1H)

#=====95%Cl with Least Square =============

Model2.1H.grid=ref.grid(Model2.1H)

Model2.1H.grid

summary(Model2.1H.grid)

lsmeans(Model2.1H.grid, ~ PERANTARA)

A=lsmeans(Model2.1H.grid, ~ PERANTARA)

PerantaraIn <- lsmeans(Model2.1H, ~ PERANTARA)

ls=contrast(PerantaraIn, "trt.vs.ctrl")

contrast(PerantaraIn, "trt.vs.ctrl")

confint(ls,adjust = "bonferroni")

Example R Script for Secondary Outcomes

Outcome: VAS (Visual Analogue Scale)

Level 1: Measurement Time Points (MTPs)*PERANTARA and Baseline as the Covariate

Level 2: Participants

Level 3: Hospital

R Script

library(lme4)

#model1 added fixed effect level 1 with two levels

Model1<- lme(fixed = VAS ~ MTP*PERANTARA + VAS_0, random = ~1|PARTICIPANTS , data = Data)

summary (Model1)

#model1 added fixed effect level 1 with three levels

Model1H<- lme(fixed = VAS ~ MTP*PERANTARA + VAS_0, random = ~1|Hospital/PARTICIPANTS , data = Data)

summary (Model1H)

#deciding Best Model

anova(Model1,Model1H)

#=====95%Cl with Least Square =============

library(lsmeans)

Model1ls<- lme(fixed = VAS ~ MTP*PERANTARA + VAS_0, random = ~1|Hospital/PARTICIPANTS , data = Data)

summary(Model1ls)

Model1.grid=ref.grid(Model1ls)

Model1.grid

summary(Model1.grid)

lsmeans(Model1.grid, ~PERANTARA|MTP)

PerantaraIn <- lsmeans(Model1.grid, ~PERANTARA|MTP)

ls=contrast(PerantaraIn, "trt.vs.ctrl")

confint(ls,adjust = "bonferroni")
